# A Transcription Factor-Based Risk Model for Predicting the Prognosis of Prostate Cancer and Potential Therapeutic Drugs

**DOI:** 10.1155/2021/6894278

**Published:** 2021-11-22

**Authors:** Ruixiang Luo, Mengjun Huang, Yinhuai Wang

**Affiliations:** Department of Urology, The Second Xiangya Hospital, Central South University, Changsha, Hunan 410011, China

## Abstract

**Background:**

Prostate cancer (PC) is one of the most critical cancers affecting men's health worldwide. The development of many cancers involves dysregulation or mutations in key transcription factors. This study established a transcription factor-based risk model to predict the prognosis of PC and potential therapeutic drugs.

**Materials and Methods:**

In this study, RNA-sequencing data were downloaded and analyzed using The Cancer Genome Atlas dataset. A total of 145 genes related to the overall survival rate of PC patients were screened using the univariate Cox analysis. The Kdmist clustering method was used to classify prostate adenocarcinoma (PRAD), thereby determining the cluster related to the transcription factors. The support vector machine-recursive feature elimination method was used to identify genes related to the types of transcription factors and the key genes specifically upregulated or downregulated were screened. These genes were further analyzed using Lasso to establish a model. Gene Ontology (GO) and Kyoto Encyclopedia of Genes and Genomes (KEGG) were used for the functional analysis. The TIMER algorithm was used to quantify the abundance of immune cells in PRAD samples. The chemotherapy response of each GBM patient was predicted based on the public pharmacogenomic database, Genomics of Drug Sensitivity in Cancer (GDSC, http://www.cancerrxgene.org). The R package “pRRophetic” was applied to drug sensitivity (IC50) value prediction.

**Results:**

We screened 10 genes related to prognosis, including eight low-risk genes and two high-risk genes. The receiver operating characteristic (ROC) curve was 0.946. Patients in the high-risk score group had a poorer prognosis than those in the low-risk score group. The average area under the curve value of the model at different times was higher than 0.8. The risk score was an independent prognostic factor. Compared with the low-risk score group, early growth response-1 (*EGR1)*, *CACNA2D1*, *AC005831.1*, *SLC52A3*, *TMEM79*, *IL20RA*, *CRACR2A*, and *FAM189A2* expressions in the high-risk score group were decreased, while *AC012181.1* and *TRAPPC8* expressions were increased. GO and KEGG analyses showed that prognosis was related to various cancer signaling pathways. The proportion of B_cell, T_cell_CD4, and macrophages in the high-risk score group was significantly higher than that in the low-risk score group. A total of 25 classic immune checkpoint genes were screened out to express abnormally high-risk scores, and there were significant differences. Thirty mutant genes were identified; in the high- and low-risk score groups, *SPOP*, *TP53*, and *TTN* had the highest mutation frequency, and their mutations were mainly missense mutations. A total of 36 potential drug candidates for the treatment of PC were screened and identified.

**Conclusions:**

Ten genes of both high-and low-risk scores were associated with the prognosis of PC. PC prognosis may be related to immune disorders. *SPOP*, *TP53*, and *TTN* may be potential targets for the prognosis of PC.

## 1. Introduction

Prostate cancer (PC) is the second most common cause of cancer-related mortality in men in developed countries [1]. In the past decades, PC treatments have mainly included surgery, androgen deprivation therapy (ADT), radiotherapy (RT), ablation therapy, chemotherapy, and emerging immunotherapy [2]. Although the latest advances in PC therapy have significantly improved patient prognosis, advanced PC is still associated with higher morbidity and mortality [3]. Therefore, there is an urgent need to explore novel and accurate biomarkers to assess the diagnosis and prognosis of patients with PC. This study aimed to predict the prognosis of PC and potential therapeutic drugs based on a risk model of transcription factors.

In recent years, people have become increasingly interested in incorporating genomic information into risk models, which aggregates the effects of many genetic variations in the entire human genome into a score, and these models have been proved to have prognostic value in a variety of diseases, including PC [4]. Multivariate risk assessment has been used as a powerful tool for PC diagnosis [5].

Transcription factors play a vital role in regulating metabolism, immunity, and tumorigenesis [6]. Cancer progression is closely related to transcription factors that regulate cell proliferation, differentiation, apoptosis, angiogenesis, inflammation, and the immune response [7]. These factors may affect cancer progression through protein-protein interactions and the transcriptional regulation of signaling pathways [8]. Previous studies have shown that mutated or dysregulated transcription factors affect cancer development, and targeted transcription factor activity may affect cancer treatment [9, 10].

The expression imbalance or mutation of key transcription factors also has an impact on the development, treatment, and prognosis of PC [11]. Pan et al. have shown that c-Myc can regulate the expression of FoxM1 by directly binding to its promoter, thereby regulating the proliferation and invasion of PC cells [12]. Transcription factors affect drug resistance, metastasis, and immunosuppression of cancer drugs and are expected to become new targets for anti-PC drug therapy [13]. The androgen receptor (AR) is a type I nuclear hormone receptor and the main drug target for PC [14]. Previous studies have shown that multiple transcription factors are closely related to AR in PC [15].

Studies have shown that immune cell infiltration is related to the prognosis of patients with PC [16]. Various immune cells, including tumor-associated neutrophils, tumor-infiltrating macrophages, suppressor cells, and mast cells of myeloid origin, promote PC through various intercellular signaling pathways [17]. For example, M2 macrophages and regulatory T cells (Tregs) can promote cancer progression by suppressing antitumor immune responses [18]. Transcription factors induce the conversion of macrophages from M1 to M2 type in the microenvironment of PC and promote the progression of PC [19].

In this study, we established a risk score model through a Lasso analysis. The correlation between the risk score and PC prognosis-related gene expression was further explored. Gene ontology (GO) and Kyoto encyclopedia of genes and genomes (KEGG) predicted the main signaling pathways involved in the risk score model and prognosis-related genes. The correlation between the risk score model, immune cells, and gene mutations was discussed. Furthermore, potential drugs for PC treatment were predicted. These results may provide a theoretical basis for exploring the molecular mechanisms and prognostic markers of PC.

## 2. Materials and Methods

### 2.1. Data Set and Preprocessing

The Cancer Genome Atlas (TCGA) prostate adenocarcinoma (PRAD) dataset was downloaded from UCSC Xena [20]. RNA-sequencing (RNA-seq) data were downloaded from the TCGA data portal. Fragments Per Kilobase of exon model per Million mapped fragments (FPKM) was then converted to Transcripts Per Kilobase of exon model per Million mapped reads (TPM). TPM standardizes expression levels between transcriptome samples and is a common data format.

### 2.2. PRAD Clustering Based on Transcription Factors

A total of 1665 transcription factors were obtained from the Animal TFDB database [21]. The intersection of genes with TCGA yielded 1649 genes. Then, 145 genes were screened using the univariate Cox analysis. The Kdmist clustering method was used to classify PRAD, thereby determining the cluster related to the transcription factors.

### 2.3. Establishment of Risk Score Based on Transcription Factors

To further establish a prognostic score based on the transcription factor cluster, we performed a differential analysis of the two transcription factor clusters and obtained 1944 genes. Fifty-four genes were screened by univariate analysis. The support vector machine (SVM)-recursive feature elimination method was used to identify thirty-four genes related to the types of transcription factors. The selection operator (Lasso) Cox model was further screened using the R package glmnet, with *p* < 0.05 transcription factor types associated with genes [22]. The risk score was defined as the sum of the gene expression value × Lasso coefficient. The risk scoring formula was as follows: risk score = −0.2435 × Early growth response-1 (*EGR1)* + −0.226 × *CACNA2D1* + 0.9155 × *AC012181.1* + −0.5831 × *.1* + 1.5148 × *TRAPPC8* + −0.4412 × *SLC52A3* + −0.5652 × *TMEM79* + −0.151 × *IL20RA* + −0.0269 × *CRACR2A* + −0.2343 × *FAM189A2*. The median of the transcriptional factor risk score was used to divide the patients into high-and low-risk score groups.

### 2.4. Pathway and Immune Infiltration Analysis

The differences between the two groups of expression matrices, the high- and low-risk score groups, were analyzed. The standard of differential gene expression was |log2 fold change (FC)| > 1.5 and *p* < 0.05. GO annotation and KEGG pathway enrichment analysis were performed using the Database for Annotation, Visualization, and Integrated Discovery (DAVID, https://david.ncifcrf.gov/) online tool. GO included cellular component (GO CC), molecular function (GO MF), and biological process (GO BP) analysis. The TIMER algorithm was used to quantify the abundance of immune cells in the PRAD samples (29092952). TIMER was used to reanalyze gene expression data from TCGA, which included 10,897 samples from 32 cancer types, to estimate the abundance of six subtypes of tumor-infiltrating immune cells, including T_ cell CD4+, T_ cell CD8+, B_ cell, macrophages, dendritic cells, and neutrophils. We downloaded the immune invasion levels of the patients with PC. The differences in immune infiltration between the high-and low-risk score groups were compared.

### 2.5. Mutation Analysis

The gene mutations of the two groups of transcription factor risk scores were compared. The Maftools package [23] helped visualize the TCGA-AML mutation data, and the genetic mutation patterns were examined in both groups.

### 2.6. Statistical Analysis

The Shapiro-Wilk normality test was used to test the normality of the variables. For normally distributed variables, the unpaired Student's *t*-test was used to compare the differences between the two groups. The Wilcoxon test was used to compare the nonnormally distributed variables. The data were mainly visualized using the R package ggplot2. In the analysis of differentially expressed genes, we used the Benjamini-Hochberg method. |log2 FC| >1 and false discovery rate (FDR) < 0.05 was set as the threshold [24]. This method converted the *p* value to FDR to identify the important genes. The Kaplan-Meier method was used to generate and visualize the survival curves of the subgroups. The log-rank test was used to determine the statistical significance of the differences in each dataset. All survival curves were generated using the R package survminer. Receiver operating characteristic (ROC) curve analysis and area under the curve (AUC) were used to assess the predictive performance of the risk models and their differential transcription factors using the R package pROC. Volcano maps and heat maps were generated from the gplots and heatmap packages in the edgeR package. All statistical analyses were performed using R 3.5.1. All the tests were two-sided, and statistical significance was set at *p* < 0.05.

## 3. Results

### 3.1. Transcription Factor Clustering

**Figure 1:**
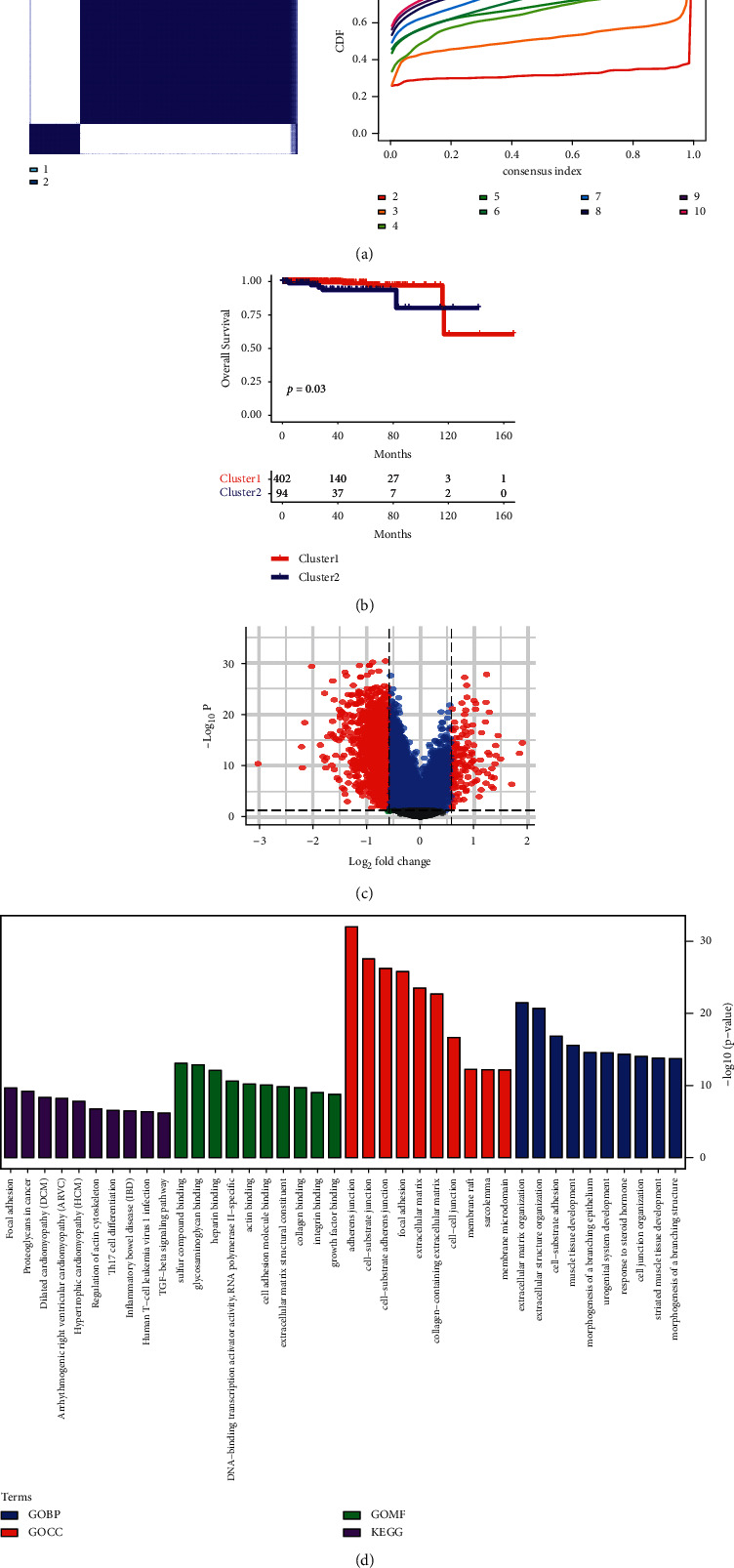
PRAD clustering and analysis. (a) Kdmist clustering. (b) Survival analysis. *p* < 0.05 indicated that there was a significant difference in the survival rate between the two groups. (c) Volcano map. (d) Functional analysis. PRAD, prostate adenocarcinoma.

Studies have shown that an imbalance in transcription factors can damage the normal prostate transcription network and lead to the progression of malignant diseases [25]. To explore the relationship between transcription factors and PC, we clustered PRAD into two categories based on the transcription factors (Figures 1(a) and 1(b)). There was a significant difference in the survival analysis of the two categories (*p*=0.03) (Figure 1(b)). The volcano map showed the distribution of the two types of differential genes (Figure 1(c)). Functional analysis was performed for the two types of differential genes, including GO BP, GO CC, GO MF, and KEGG (Figure 1(d)). GO BP showed that the two types of differential genes were mainly involved in regulating focal adhesion, proteoglycans in cancer, dilated cardiomyopathy (DCM), arrhythmogenic right ventricular cardiomyopathy (ARVC), hypertrophic cardiomyopathy (HCM), regulating actin cytoskeleton, Th17 cell differentiation, inflammatory bowel disease (IBD), human T-cell leukemia virus 1 infection, and the TGF-beta signaling pathway. GO CC showed that the two types of differential genes were mainly involved in regulating sulfur compound binding, glycosaminoglycan binding, heparin binding, DNA-binding transcription activator activity, RNA polymerase II-specific, actin binding, cell adhesion molecule binding, extracellular matrix structural constituent, collagen binding, integrin binding, and growth factor binding. GO MF showed that the two types of differential genes are mainly involved in regulating adherens junctions, cell-substrate junctions, cell-substrate adherens junctions, focal adhesion, extracellular matrix, collagen-containing extracellular matrix, cell-cell junction, membrane raft, sarcolemma, and membrane microdomain. KEGG analysis showed that the two types of differential genes were mainly involved in regulating extracellular matrix organization, extracellular structure organization, cell-substrate adhesion, muscle tissue development, morphogenesis of a branching epithelium, urogenital system development, response to steroid hormones, cell junction organization, striated muscle tissue development, and morphogenesis of a branching structure. In addition, we also performed gene sets enriched in immune and functional analyses on transcription factor-based clustering (GSEA). As shown in Table 1 and Figure S3, the top six low *p* value data sets include apoptosis, T-cell receptor signaling pathway, natural killer cell-mediated cytotoxicity, meiosis chromosome separation, regulation of autophagy, and regulation of chromosome separation. Hierarchical clustering analysis showed that the two types of differential genes may be closely related to the occurrence and development of PC through these pathways.

### 3.2. Establishment of Prognostic Scores Based on Transcription Factors

**Figure 2:**
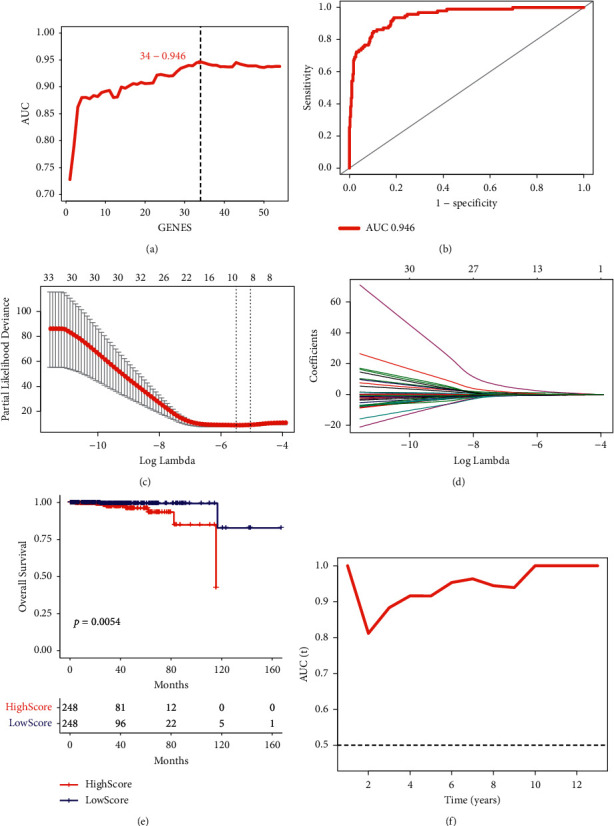
Screening of genes closely related to the prognosis of PC. (a) A different analysis of the two types of transcription factors. (b) ROC curve. (c) and (d) Lasso analysis. (e) Kaplan-Meier analysis. *p* < 0.05 indicated that there was a significant difference in survival rate between the two groups. (f) AUC value. PC, prostate cancer; ROC, receiver operating characteristic; AUC, area under the curve.

We established a prognostic score based on the transcription factors to identify the genes related to the prognosis of PC. First, the two types of transcription factor clusters were analyzed for differences, and 1,944 genes were obtained. Fifty-four genes were screened using univariate analysis, and 34 genes were obtained using the SVM method (Figure 2(a)). The model established by the SVM method reversely inferred the cluster type of transcription factors; the ROC was 0.946 (Figure 2(b)). These selected genes were analyzed by Lasso, and a risk score model containing 10 genes was obtained; they were *EGR1*, *CACNA2D1*, *AC012181.1*, *AC005831.1*, *TRAPPC8*, *SLC52A3*, *TMEM79*, *IL20RA*, *CRACR2A*, and *FAM189A2*. There were eight low-risk genes and two high-risk genes (Figures 2(c) and 2(d)). The risk score model in the TCGA survival analysis showed that patients with high-risk scores had a poor prognosis (*p*=0.0054) (Figure 2(e)). The time-dependent ROC chart showed that the AUC value of the model at different times was relatively high, indicating that the model was relatively more accurate and had relatively stronger applicability (Figure 2(f)). To improve the credibility of the risk score model, we have verified the model with the GSE16560 data set. Similarly, the risk score model in the GSE16560 survival analysis showed that patients with high-risk scores had a poor prognosis, *p*=0.028 (Figure S2). We identified 10 genes that were closely related to the prognosis of PC.

### 3.3. Functional Analysis of Risk Scoring

**Figure 3:**
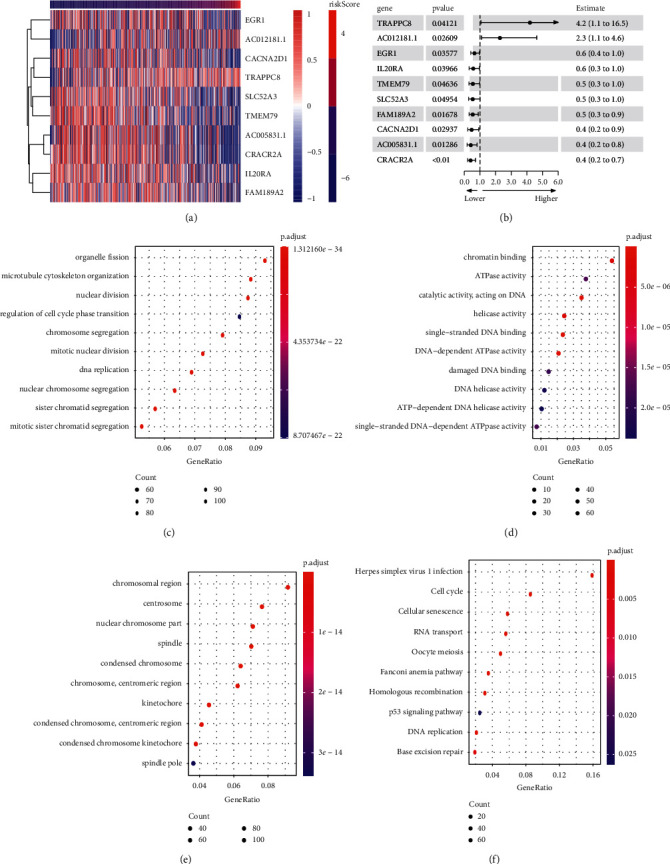
Correlation analysis of genes closely related to the prognosis of PC with risk score model. (a) Heat map. (b) Univariate factor analysis. (c) Analysis of related gene enrichment function.

Next, we explored the correlation between the risk score and gene expression. The heat map showed that the expressions of *EGR1*, *CACNA2D1*, *AC005831.1*, *SLC52A3*, *TMEM79*, *IL20RA*, *CRACR2A*, and *FAM189A2* in the high-risk score group were reduced, while *AC012181*.1 and *TRAPPC8* expressions were increased (Figure 3(a)). The univariate factor results of 10 genes in the risk score model showed *p* < 0.05. The results showed that 10 genes were significantly associated with PC prognosis (Figure 3(b)). The risk score and all genes underwent correlated analysis, correlation coefficient|>0.3-, and *p* < 0.05-related gene enrichment functions were analyzed, including GO BP (Figure 3(c)), GO MF (Figure 3(d)), GO CC (Figure 3(e)), and KEGG (Figure 3(f)). The top 10 GO terms and KEGG pathways were identified. From the biological process, it has been observed that genes related to the prognosis of PC were significantly enriched in organelle fission, microtubule cytoskeleton organization, nuclear division, regulation of cell cycle phase transition, and chromosome segregation. Chromatin binding, ATPase activity, catalytic activity acting on DNA, helicase activity, and single-stranded DNA binding were the five most important categories of molecular functions of genes related to the prognosis of PC. In terms of cell composition, genes related to the prognosis of PC were most abundant in chromosomal regions, centrosomes, nuclear chromosomes, spindles, and condensed chromosomes. KEGG analysis showed that the prognosis-related genes of PC were associated with herpes simplex virus 1 infection, cell cycle, cellular senescence, RNA transport, and oocyte meiosis. In summary, PC prognosis was significantly correlated with 10 genes and was associated with various cancer signals.

### 3.4. Immune Infiltration and Immune Checkpoint Analysis of Risk Score Model

**Figure 4:**
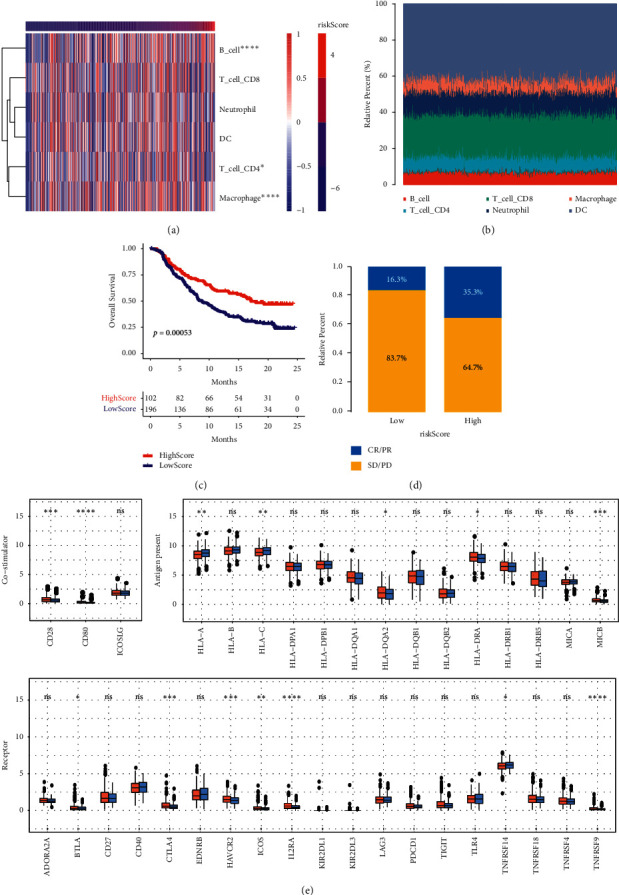
The correlation between risk score model and immune cells. (a) Heat map. (b) The proportion of cells in each sample. (c) Survival analysis. *p* < 0.05 indicated that there was a significant difference in the survival rate between the two groups. (d) The relative percentage of CR/PR and SD/PD in the high- and low-risk score. (e) Analysis of immune checkpoints at different levels. ^*∗*^*p* < 0.05. ^*∗∗*^*p* < 0.01. ^*∗∗∗*^*p* < 0.001. ^*∗∗∗∗*^*p* < 0.0001. CR, complete response; PR, partial response; PD; SD.

The above results showed that 10 genes had significant differences in the risk score. Next, we analyzed the immune infiltration and immune checkpoints of the risk score model. The heat map showed that the percentages of B_cell, T_cell_CD4, and macrophages increased significantly in the high-risk score group (Figure 4(a)).  Figure 4(b) shows that, in each sample, the proportion of dendritic cells, T_cell_CD8, and neutrophil cells ranked among the top three. In the immunotherapy data set (IMvirgor210), it was verified that high scores had a good prognosis, and low scores had a poor prognosis (*p* < 0.001; Figure 4(c)). The complete response/partial response (CR/PR) ratio of the high-risk score group was higher than that of the low-risk score group (Figure 4(d)). The above results indicated that the higher the risk score, the better the prognosis of PC, which was mainly manifested by a significant increase in the proportion of B_cell, T_cell_CD4, and macrophage cells. TCGA PRAD data showed the expression of high-and low-risk immune checkpoints (Figure 4(e), Figure S1). At the costimulator level, the expression of CD28 and CD80 was decreased in the high-risk score group. At the antigen level, the expression of HLA-A and HLA-C was increased in the high-risk score group, while the expression of HLA-DQA2, HLA-DRA, and MICB was decreased. At the receptor level, the expression of BTLA, CTLA4, HAVCR2, ICOS, IL2RA, and TNFRSF9 was decreased in the high-risk score group, while the expression of TNFRSF14 was increased. At the ligand level, the expression of CD40LG, CXCL9, CXCL10, IFNG, IL10, and TNFSF9 was reduced in the high-risk score group. At the cell adhesion level, there was no statistically significant difference between the high- and low-risk scores. On the other hand, the expression of ARG1, ENTPD1, GZMA, HMGB1, and IDO1 was decreased in the high-risk score group. The abnormal expression of the above classic immune checkpoint genes showed significant differences in the risk score. In summary, immune infiltration and immune checkpoints were significantly correlated with high-and low-risk scores.

### 3.5. Gene Mutation Analysis Based on Risk Score

**Figure 5:**
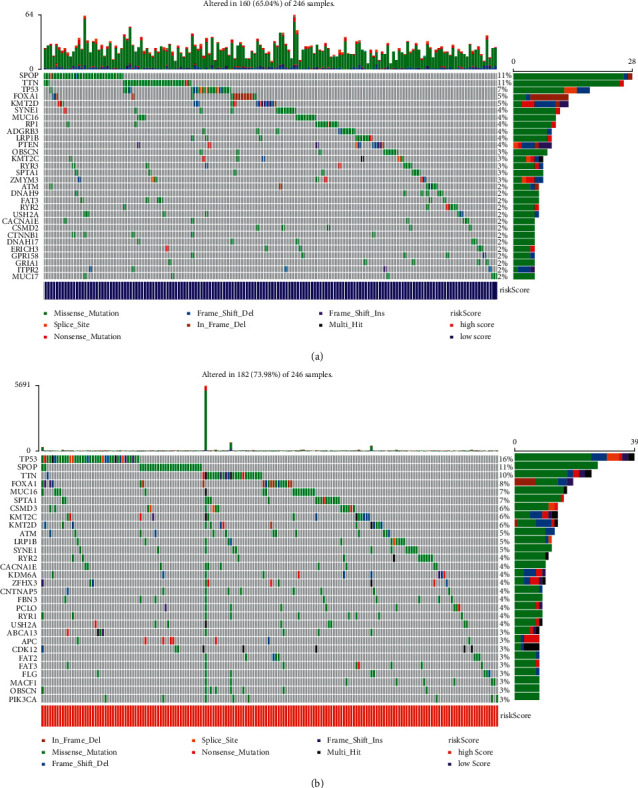
The waterfall chart showed the top 30 mutant genes. (a) Low-risk score group. (b) High-risk score group.

Next, we visualized the TCGA-AML mutation data. The waterfall chart shows the mutation of genes in the low-risk score group (Figure 5(a)). In Figure 5(b), the waterfall chart showed mutations in the high-risk score group. The results showed that *SPOP*, *TP53*, and *TTN* had the highest mutation frequency in the high- and low-risk score groups, and the mutations were mainly missense mutations.

### 3.6. Drug Analysis

**Figure 6:**
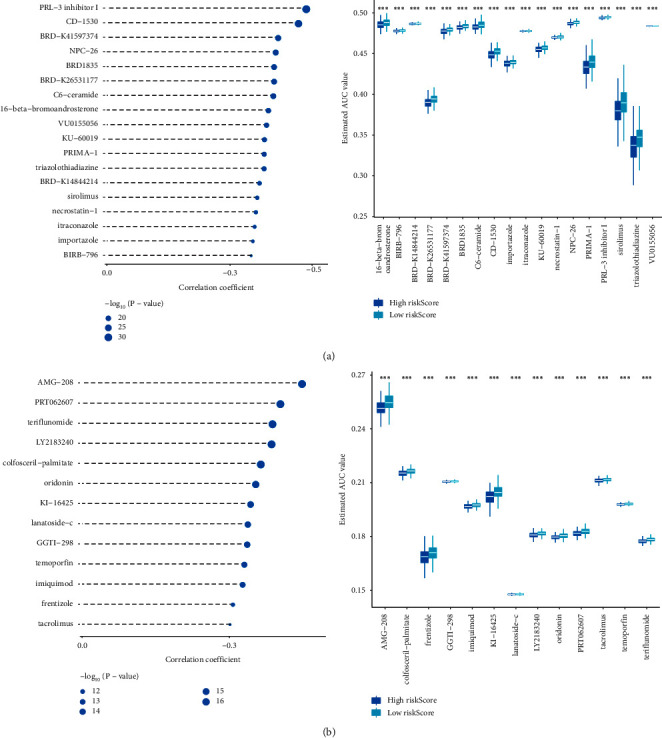
Drug candidates for the treatment of PC. (a) CTRP. (b) PRISM. ^*∗*^*p* < 0.05. ^*∗∗*^*p* < 0.01. ^*∗∗∗*^*p* < 0.001. ^*∗∗∗∗*^*p* < 0.0001. PC, prostate cancer.

Potential therapeutic agents in the TCGA subgroup were predicted based on the drug sensitivity AUC values of CTRP2.0 (Figure 6(a)) and PRISM (Figure 6(b)), as well as CCLE expression profile data [26]. The first 18 important drugs in CTRP2.0 were PRL-3 inhibitor 1, CD-1530, BRD-K41597374, NPC-26, BRD 1835, BRD-K26531177, C6-ceramide, 16-beta-bromoandrosterone, VU0155056, KU- 60019, PRIMA-1, triazolothiadiazine, BRD-K14844214, sirolimus, necrostatin-1, itraconazole, importazole, and BIRB-796. There were significant differences between the above drugs in the high- and low-risk score groups. The top 18 most important drugs in PRISM were AMG-208, PRT062607, teriflunomide, LY2183240, colfosceril-palmitate, oridomin, KI-16425, lanatoside-c, GGTI-298, temoporfin, imiquimod, rentizole, and tacrolimus. The above drugs in the high- and low-risk score groups were significantly different. These drugs may be effective anti-PC drugs in the future.

## 4. Discussion

PC is one of the most common cancers in men worldwide [27]. Its prognostic variables are beneficial for clinical trial design and treatment strategies [28]. In this study, a risk model based on transcription factors predicted the prognostic biomarkers of PC and potential therapeutic drugs.

The dysregulation of transcription factors is an important driving force in tumorigenesis [29]. EGR1 is a transcription factor associated with PC [23]. Bioinformatics analysis indicated that EGR1 may play an important role in the pathogenesis and progression of PC [30]. The transcriptomic analysis confirmed that TMEM79 is a diagnostic marker of PC [31]. TMEM79 has been identified as a potential biomarker for PC by deep sequencing [32]. A recent study showed that epigenetic changes in *CRACR2A* are related to the lethal progression of PC metastasis [33]. In this study, we first grouped PRAD into two categories based on transcription factors. There was a statistical difference in the survival analysis of the two categories, *p*=0.03. The volcano map showed the distribution of two types of differential genes. Next, we established a prognostic score based on transcription factors to obtain a risk score model containing ten genes. Compared with the low-risk score group, EGR1, CACNA2D1, AC005831.1, SLC52A3, TMEM79, IL20RA, CRACR2A, and FAM189A2 expressions in the high-risk score group were decreased, while AC012181.1 and TRAPPC8 expressions were increased. Our results were consistent with previous studies. At present, most of the research on the role of *CACNA2D1*, *SLC52A3*, *IL20RA*, *FAM189A2*, and *TRAPPC8* in PC is lacking. However, they have important prognostic significance for other cancers [34–38]. Therefore, further single-gene bioinformatics analyses are required. However, the two genes *AC005831.1* and *AC012181.1* have not yet been referenced, and there is still great research significance. Among the 10 screened genes, there were eight low-risk genes and two high-risk genes. Ten genes had statistical differences in the risk score. Patients with a high-risk score had worse overall survival. Previous studies have shown that *EGR1*, *TMEM79*, and *CRACR2A* are closely related to the progression of a variety of cancers, and it is worthwhile to further explore their molecular mechanisms.

It has been reported that transcription factors activate the NOTCH pathway to promote the growth and invasion of prostate cancer cells [39]. The transcription factor Sp3 regulates BNIP3 to inhibit the proliferation of prostate cancer cells and cause apoptosis [40]. In this study, we further performed two types of differential gene function analysis. The results showed that transcription factors were involved in PC prognosis by regulating cancer signal pathways, mainly involving extracellular matrix organization, extracellular structure organization, cell-substrate adhesion, muscle tissue development, and morphogenesis of a branching epithelium. Based on GSEA, transcription factors were mainly involved in apoptosis, T-cell receptor signaling pathway, natural killer cell-mediated cytotoxicity, meiosis chromosome separation, regulation of autophagy, and regulation of chromosome separation. Our results indicated that transcription factors might affect PC prognosis through immunity and cancer signaling pathways.

The activation and recruitment of immune cells during inflammation lead to a cellular environment rich in cytokines and chemokines, thereby affecting the development of PC [41]. Previous studies have shown that M0 macrophages are associated with the prognosis of PC [42]. It was reported that reduced infiltration of CD8 + T cells and monocytes, as well as the increased activation of natural killer cells and naive B cells, may also be associated with the prognosis of PC [43]. Similarly, our data showed that PC prognosis was related to B_cell, T_cell_CD4, and macrophages. In the immune-associated risk model, the prognosis of osteosarcoma patients with a high score was significantly improved, whereas, in the immune-associated gene risk model, the prognosis of patients with a high score was poor [44]. In this study, PC patients with high risk had a poorer prognosis in a risk score model based on transcription factors. Interestingly, the risk score in the immunotherapy dataset showed that patients with low-risk scores had poor overall survival. We mainly expounded from two aspects. On the one hand, it could be caused by different tumors. On the other hand, the number of samples in the TCGA queue was relatively small, which may lead to some bias. This is a limitation of our study. The results showed that increased B_cell, T_cell_CD4, and macrophages were associated with an improvement in the prognosis of PC patients. It has been reported that a human monoclonal antibody to human *αν* integrins (intetumumab) evaluates PC progression through CR/PR [45]. In this study, the data showed that the CR/PR ratio of the high-risk score group was higher than that of the low-risk score group. Our results were consistent with previous results. A cohort study showed that immune checkpoint-related proteins, including BTLA, CD28, and CD80, can predict the prognosis of PC [46]. Our data showed that the expression of CD28 and CD80 was decreased in the high-risk score group, which indicated that reduced CD28 and CD80 predicted a better prognosis for PC. The enrichment of IDO-1 immune checkpoints has been identified in nondegeneration PCs, providing a new therapeutic target for patients with bone metastases [47]. In this study, the data showed that IDO1 expression was decreased in the high-risk score group. Our results also indicated that IDO1 enrichment predicted a poorer PC prognosis. Besides, we also found that the expression of HLA-A, HLA-C, and TNFRSF14 was increased in the high-risk score group, while HLA-DQA2, HLA-DRA, MICB, BTLA, CTLA4, HAVCR2, ICOS, IL2RA, TNFRSF9, CD40LG, CXCL9, CXCL10, IFNG, IL10, TNFSF9, ARG1, ENTPD1, GZMA, and HMGB1 expression was decreased. We clarified for the first time that the PC prognosis might be related to the above-mentioned immune checkpoints, which may provide a new direction for PC treatment. Our results indicated that there were significant differences between immune infiltration and immune checkpoints in the risk score model.

Bioinformatics analysis identified an increased risk of recurrence in patients with PC with *TP53* mutations [48]. Previous studies have shown that *SPOP* is the most commonly mutated gene in PC [49]. Our data showed that *SPOP* and *TP53* had the highest mutation frequency in the high- and low-risk score groups. *SPTA1*, *ATM*, *FOXA1*, *CSMD3*, and *LRP1B* are commonly mutated genes in PC [50]. Our results are consistent with those of previous studies. In addition, we found that *TTN* had a high mutation frequency in the high- and low-risk groups, and it had a high mutation frequency in bladder cancer [51]. *TTN* may be a new target that affects the prognosis of PC. At present, research on the four drug candidates, PRL-3 inhibitor 1, NPC-26, AMG-208, and PRT062607, for PC treatment is a novel topic; nevertheless, studies have shown that these candidates are targets of anticancer drugs [52–55]. Therefore, the roles of PRL-3, NPC-26, AMG-208, and PRT062607 in PC are worth further investigation. In addition, we also found 32 potential drugs that have not been studied so far, which are CD-1530, BRD-K41597374, BRD 1835, BRD-K26531177, C6-ceramide, 16-beta-bromoandrosterone, VU0155056, KU-60019, PRIMA -1, triazolothiadiazine, BRD-K14844214, sirolimus, necrostatin-1, itraconazole, importazole, BIRB-796, teriflunomide, LY2183240, colfosceril-palmitate, oridomin, KI-16425, lanatoside-c, GGTI-298, temo, rentizolefin, imiquimod, tacrolimussirolimus, necrostatin-1, itraconazole, importazole, and BIRB-796. The above drugs were significantly increased in the high-risk score group, indicating a better prognosis for PC. The therapeutic effects of 36 potential drugs in PC need to be further explored.

## 5. Conclusion

The prognosis of PC patients was likely to be related to abnormal expression of EGR1, CACNA2D1, AC005831.1, SLC52A3, TMEM79, IL20RA, CRACR2A, FAM189A2, AC012181.1, and TRAPPC8. The worsening condition of PC patients may cause immune dysregulation. *SPCP*, *TP53*, and *TTN* may be potential targets for the prognosis of PC. We screened 36 candidate drugs for PC treatment. Our findings may provide theoretical support for the molecular mechanism and prognostic biomarkers of PC.

## Figures and Tables

**Table 1:** Gene sets enriched in immune and functional analysis on transcription factor-based clustering (GSEA).

Description	NES	*p* value
KEGG_APOPTOSIS	−1.3510	0.0002
KEGG_T_CELL_RECEPTOR_SIGNALLING_PATHWAY	−1.2701	0.0011
KEGG_NATURAL_KILLER_CELL_MEDIATED_CYTOTOXICITY	−1.2192	0.0063
GO_MEIOTIC_CHROMOSOME_SEGREGATION	1.4768	0.028
KEGG_REGULATION_OF_AUTOPHAGY	−1.2672	0.029
GO_REGULATION_OF_CHROMOSOME_SEGREGATION	1.3010	0.037

## Data Availability

The data used to support the findings of this study are available from the corresponding author upon request.

## Supplementary Materials

Supplementary Materials Supplementary Figure S1: analysis of immune checkpoints at different levels between risk score model and immune cells. ^*∗*^*p* < 0.05. ^*∗∗*^*p* < 0.01. ^*∗∗∗*^*p* < 0.001. ^*∗∗∗∗*^*p* < 0.0001. Supplementary Figure S2: the risk score model is verified by the GSE16560 data set. Supplementary Figure S3: gene sets enriched in immune and functional analysis on transcription factor-based clustering (GSEA).
