# Supplementary material for: A Transcription Factor-Based Risk Model for Predicting the Prognosis of Prostate Cancer and Potential Therapeutic Drugs
Source: Evid Based Complement Alternat Med. 2021 Nov 22;2021:6894278. doi: 10.1155/2021/6894278 (PMC8629613; doi:10.1155/2021/6894278)
Supplement: Supplementary Materials — Supplementary Figure S1: analysis of immune checkpoints at different levels between risk score model and immune cells. ∗p < 0.05. ∗∗p < 0.01. ∗∗∗p < 0.001. ∗∗∗∗p < 0.0001. Supplementary Figure S2: the risk score model is verified by the GSE16560 data set. Supplementary Figure S3: gene sets enriched in immune and functional analysis on transcription factor-based clustering (GSEA). [file 6894278.f1.docx]

## Supplementary Materials


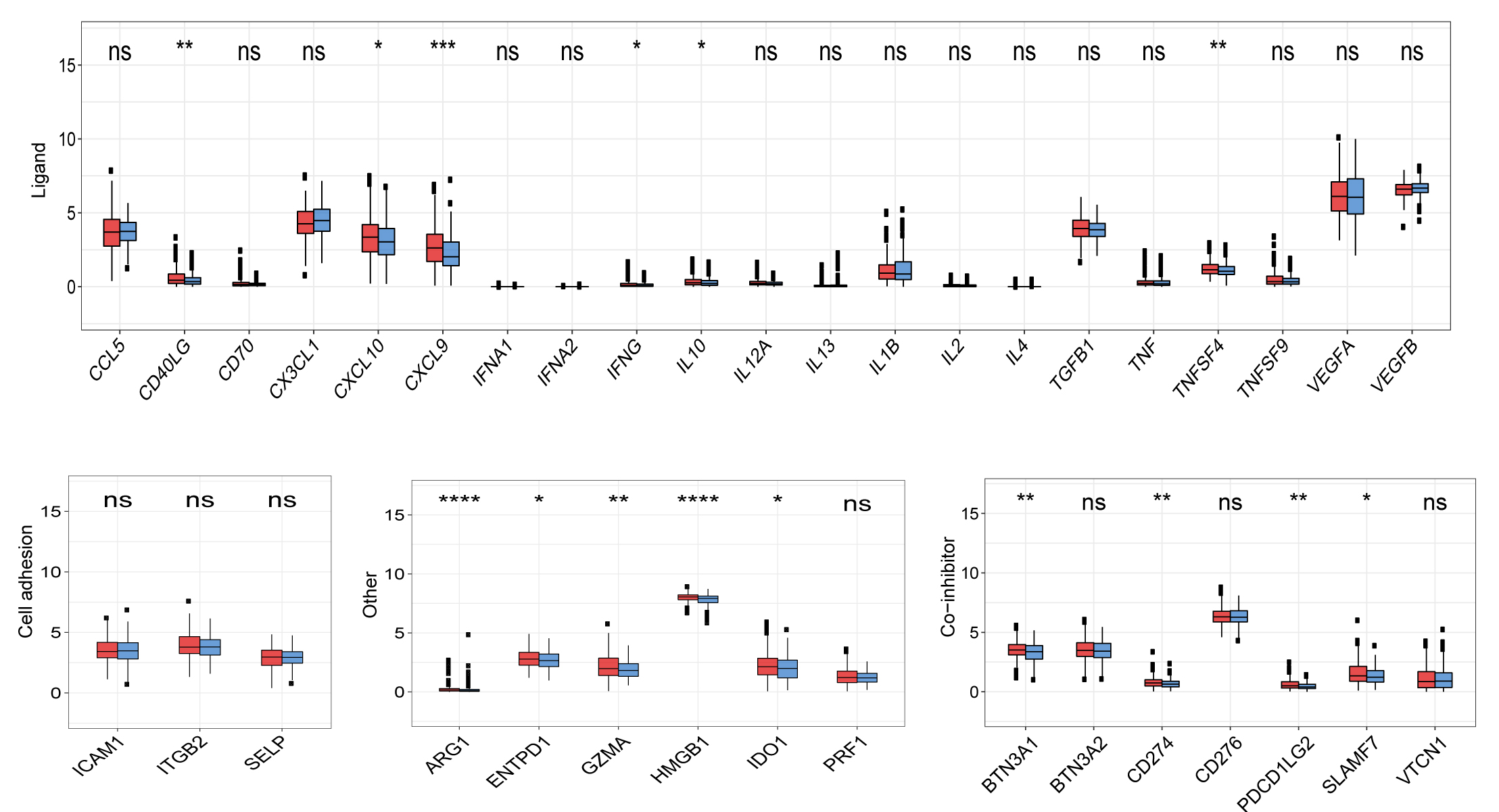


Supplementary Figure S1. Analysis of immune checkpoints at different levels between risk score model and immune cells. *, *p* < 0.05. **, *p* < 0.01. ***, *p* < 0.001. ****, *p* < 0.0001.


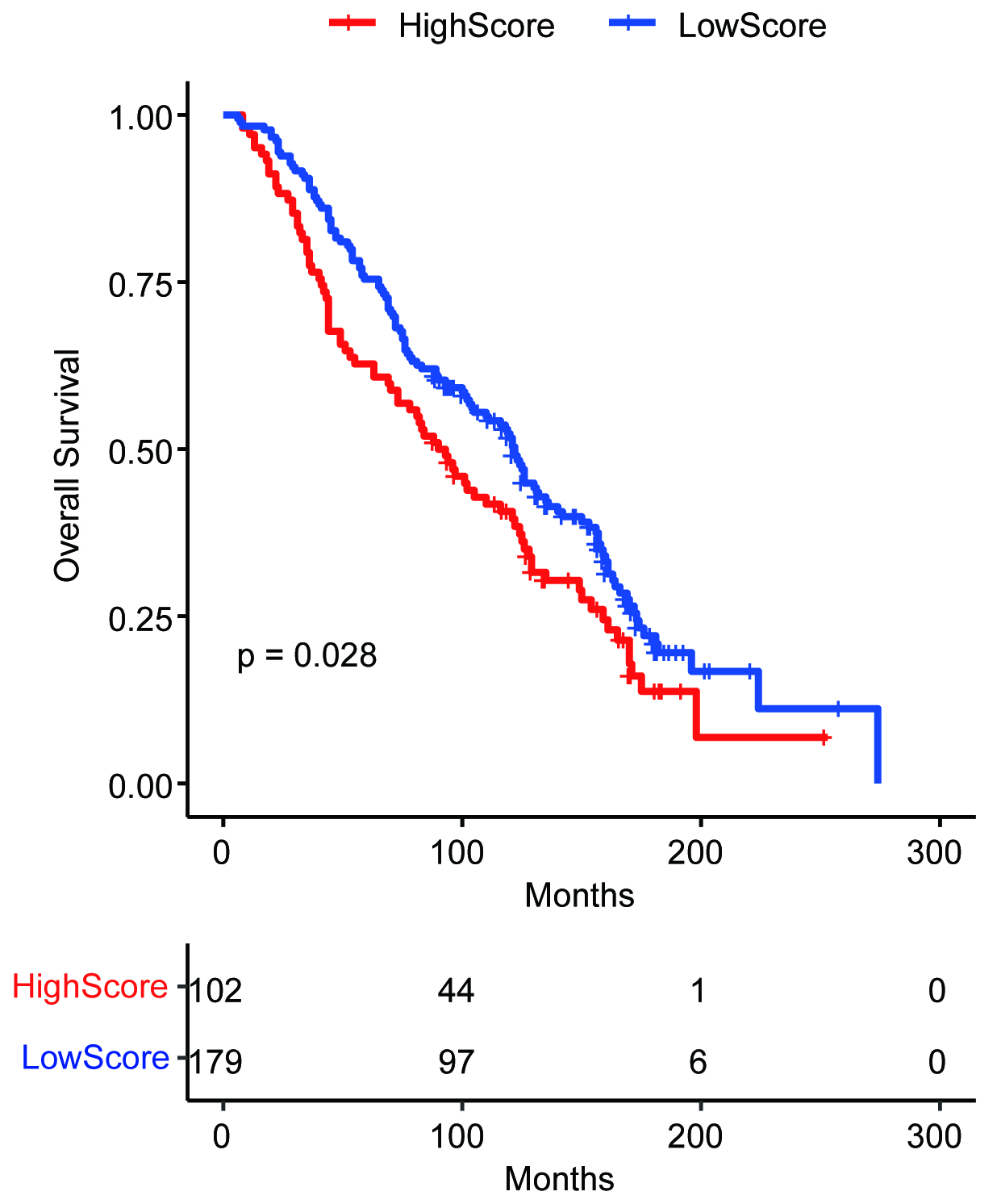


Supplementary Figure S2: The risk score model have verified by the GSE16560 data set.


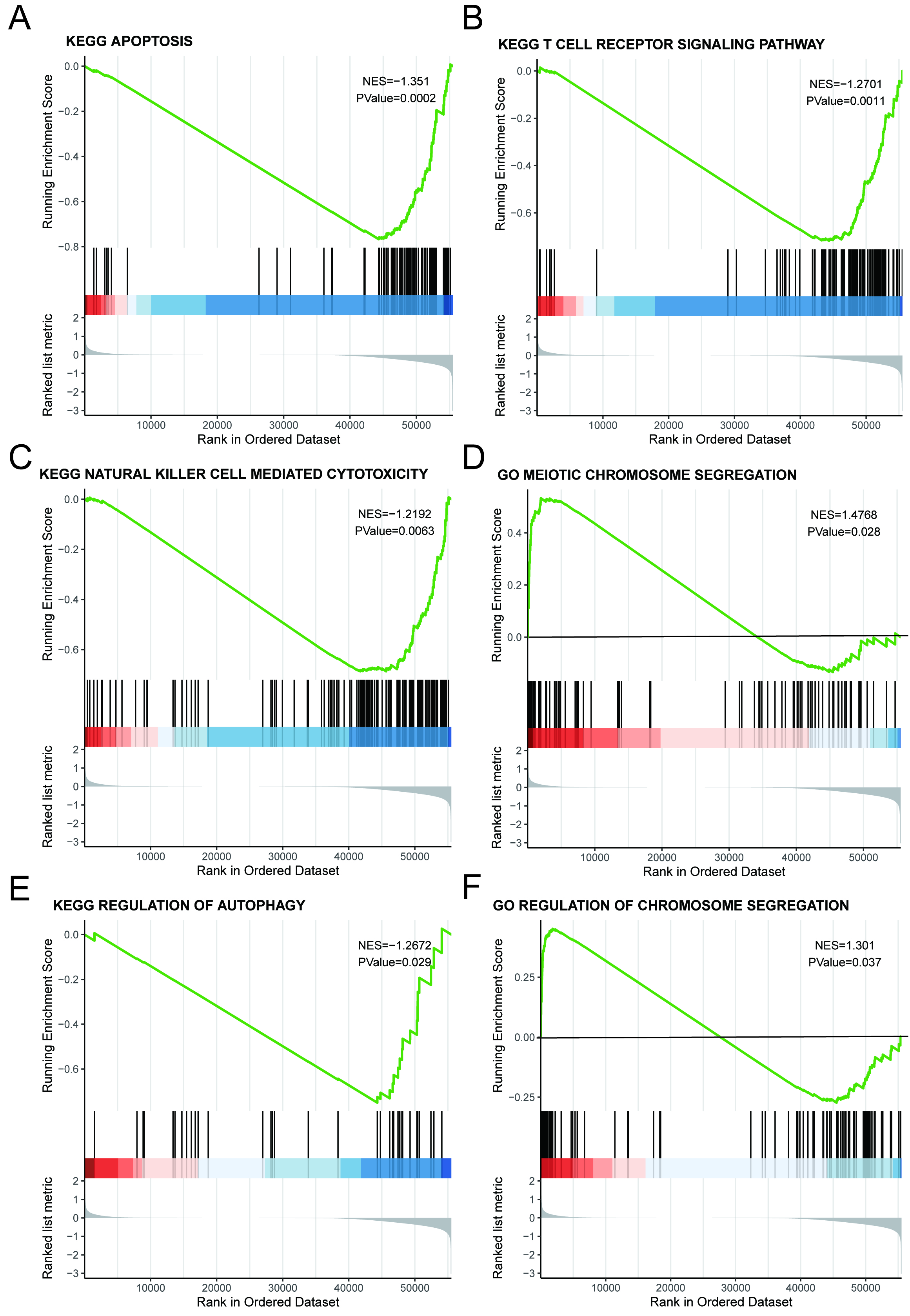


Supplementary Figure S3: Gene sets enriched in of immune and functional analysis on transcription factor-based clustering (GSEA). (A) Apoptosis. (B) T cell receptor signalling pathway. (C) Natural killer cell mediated cytotoxicity. (D) Meiosis chromosome separation. (E) Regulation of autophagy. (F) Regulation of chromosome separation. NES, normalized ES. GO, Gene ontology. KEGG, Kyoto encyclopedia of genes and genomes.
